# Enhanced Infrared Image Processing for Impacted Carbon/Glass Fiber-Reinforced Composite Evaluation

**DOI:** 10.3390/s18010045

**Published:** 2017-12-26

**Authors:** Hai Zhang, Nicolas P. Avdelidis, Ahmad Osman, Clemente Ibarra-Castanedo, Stefano Sfarra, Henrique Fernandes, Theodore E. Matikas, Xavier P. V. Maldague

**Affiliations:** 1 Department of Electrical and Computer Engineering, Computer Vision and Systems Laboratory, Laval University, 1065 av. de la Médecine, Quebec City, QC G1V 0A6, Canada; nico.avdel@gmail.com (N.P.A.); clemente.ibarra-castanedo@gel.ulaval.ca (C.I.-C.); xavier.maldague@gel.ulaval.ca (X.P.V.M.); 2 Aerospace Integration Research Centre (AIRC), College Road, Cranfield MK43 0AL, UK; 3 Department of Inspection of Components and Assemblies, Fraunhofer Institute for Nondestructive Testing IZFP, 66123 Saarbrücken, Germany; Ahmad.Osman@izfp.fraunhofer.de; 4 University of Applied Sciences, htw saar, 66117 Saarbrücken, Germany; 5 Department of Industrial and Information Engineering and Economics, Las.E.R. Laboratory, University of L’Aquila, 67100 L’Aquila, Italy; stefano.sfarra@univaq.it; 6 Tomsk Polytechnic University, 634028 Tomsk, Russia; 7 School of Computer Sciences, Federal University of Uberlandia, Uberlandia 38400-902, Brazil; henrique.fernandes@ufu.br; 8 Mechanics, Smart Sensors and Nondestructive Evaluation (MSS-NDE) Laboratory, Department of Materials Science and Engineering, University of Ioannina, 45110 Ioannina, Greece; matikas@otenet.gr

**Keywords:** infrared thermography, polynomial fitting, low-order derivative, composite, low-velocity impact

## Abstract

In this paper, an infrared pre-processing modality is presented. Different from a signal smoothing modality which only uses a polynomial fitting as the pre-processing method, the presented modality instead takes into account the low-order derivatives to pre-process the raw thermal data prior to applying the advanced post-processing techniques such as principal component thermography and pulsed phase thermography. Different cases were studied involving several defects in CFRPs and GFRPs for pulsed thermography and vibrothermography. Ultrasonic testing and signal-to-noise ratio analysis are used for the validation of the thermographic results. Finally, a verification that the presented modality can enhance the thermal image performance effectively is provided.

## 1. Introduction

Composite materials are being increasingly used in aircraft, vehicles, ships, and sports equipment thanks to their significant weight reduction at the same strength. However, the defects that are produced accidently at the manufacturing phase will affect the safety of the products. Therefore, it is important to develop the inspection techniques to assess the products. Microscopic inspection is effective, but it involves the partial or total destruction of the specimens and therefore it is not a feasible method for inline inspection [1].

Non-destructive testing (NDT) is a more practical solution than the above-mentioned destructive technique. Traditional NDT techniques include ultrasonic testing, magnetic flux leakage and X-ray computed tomography, etc. These techniques are well studied and nowadays established for quality control; however, each of them has some specific limitations for different types of defects inspection [2,3,4]. Nowadays, infrared thermography (IRT) is increasingly used and studied as an alternative or in complement to the traditional NDT techniques. This is because IRT has the advantages of fast inspection rate, being contactless, having high spatial resolution, improved acquisition rate and the development of advanced infrared image processing techniques [5]. IRT involves different thermal excitation methods such as optical excitation thermography, ultrasonic excitation, laser excitation thermography and inductive thermography, etc. [6,7,8]. Advanced signal processing plays a very important role in IRT because the raw thermal images are time-domain sequences that can be post-processed in time-domain, or transformed to another such as the frequency-domain in Fourier transform analysis [9,10].

In this paper, a pre-processing modality was presented for IRT and various cases were studied involving different defects in CFRP and GFRP specimens. These specimens were post-impacted or involved resin/preform abnormalities. Different from a signal smoothing modality, which only uses a polynomial fitting as the pre-processing method, the presented modality instead uses the low-order derivatives to pre-process the raw thermal data. Then, advanced post-processing techniques such as principal component thermography (PCT) and pulsed phase thermography (PPT) were applied on the pre-processing data. The presented infrared image processing modality was applied on both pulsed thermography (PT) and vibrothermography (VT). Ultrasonic testing (UT) and signal-to-noise ratio (SNR) analysis were used for validation purposes. Finally, the effectiveness of this modality was verified. Specifically, this modality enhances the image performance more than only using polynomial fitting as a pre-processing modality or even only using the independent derivative processing as a post-processing technique.

## 2. Materials

**Figure 1:**
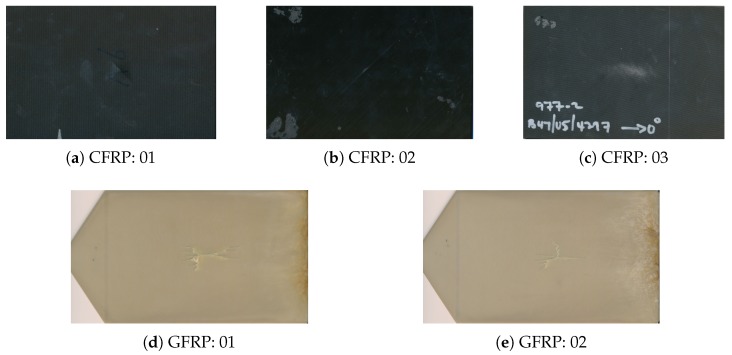
Photographs of the testing samples.

In this paper, the tested samples include three CFRPs and two GFRPs as shown in Figure 1. CFRPs 01/02 were tested with a low-velocity impact blind test. CFRP 03 included the unbalanced structural distribution of resin and preform. Their dimensions are 10 cm × 15 cm and their thickness are ∼2 mm, ∼4 mm and ∼5 mm, respectively. GFRPS 01/02 were made of Nylon matrix and short glass fiber. Their dimensions excluding the triangle heads are 12 cm × 15 cm and their thicknesses is ∼3 mm. The impact energies were 7.5 J and 6 J, respectively.

**Figure 2:**
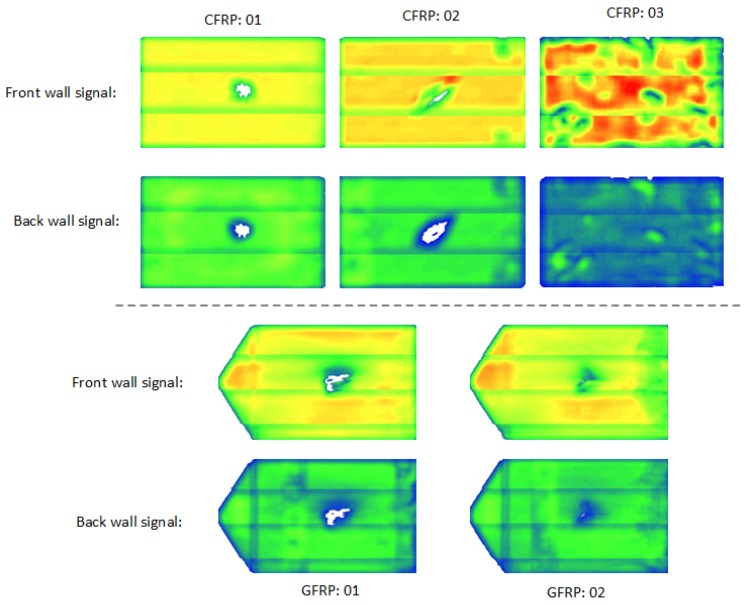
UT results.

Ultrasonic C-scan testing (UT) was first applied on the tested samples in order to find the defects prior to the infrared testing. A phased-array (PA) probe with a frequency of 2.25 MHz was used. The experiments were conducted in reflection mode and the C-scan images are shown in Figure 2. Every image is composed of three PA scanning images. The front wall reflection signal images indicate the defects near the surface, while the back wall signal images represent the defects far from the surface. In the CFRP results, the impact damage areas and resin/preform abnormalities are clearly seen. In the GFRP results, 7.5 J (GFRP 01) leads to a stronger impact damage than 6 J (GFRP 02). The UT results provide a good reference for the study of the infrared thermographic imaging approaches.

Table 1 shows the damaged areas measured in UT for the impacted specimens.

## 3. Methodology

### 3.1. Experimental Configurations

As an optical excitation thermographic approach, pulsed thermography (PT) uses high-energy lamps to generate a uniform heating on an object surface. An infrared camera is used to record the surface temperature profile. In PT, a subsurface defect causes a non-uniform cooling, which can be identified in an imaging sequence. As a time-domain sequenced imaging approach, PT allows the application of the advanced imaging processing techniques to obtain more visible imprints of the defects [9,10].

**Figure 3:**
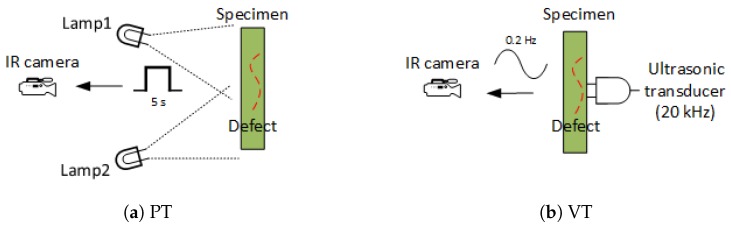
Schematic configurations.

Figure 3a shows the schematic configuration for PT. Two halogen lamps (OMNLUX PAR64, 1000 W) were used to generate the pulsed excitation signal. A mid-wave infrared camera (FLIR Phoenix, InSb, 3–5 μm, 640 × 512 pixels) at a frame rate of ∼55 Hz was used to record the temperature profile. The heating time was 5 s and the cooling time was 35 s.

As an ultrasonic excitation thermographic approach, vibrothermography (VT) was also used in this work. Different from PT, VT uses ultrasonic waves to stimulate a subsurface defect without heating the surface. The stimulation of the subsurface defect causes a complex combination of absorption, scattering, beam spreading and dispersion. The ultrasonic waves spread towards all of the orientations in the form of heat [9,10]. Figure 3b shows the schematic configuration for VT. The excitation ultrasonic waves frequency was 20 kHz and the signal modulated frequency was 0.2 Hz. The frequencies work well for a variety of applications [11]. The same infrared camera was used to record two heating/cooling circles.

### 3.2. Pre-Processing Modality

As an established image processing approach, basic thermographic signal reconstruction [12,13] has the advantage of simplicity and accuracy, and, therefore, it has been used to process thermal image sequences as an independent infrared image processing.

In this approach, a polynomial fitting is applied to the cooling sequence in the double logarithm scale. The logarithmic behavior of the time evolution exhibits a remarkable consistency in pixels. Specifically, the defect-free areas are near-linear, while the defective areas depart from the near-straight-line behavior. The logarithmic time dependence of a pixel can be approximated by a function, or a set of orthogonal functions. In most cases, a polynomial provides an excellent fit to experimental data, which is shown as follows [12]:

(1) $$ln\left[T\left(t\right)\right]=\Sigma_{n=0}^{N}a_{n}{\left[ln\left(t\right)\right]}^{n},$$

where *T* is the Temperature and *t* is the time.

A low-order expansion is applied in order to act as a low-pass filter. In the logarithmic domain, very high orders only replicate noise that appears in the low-amplitude data. Once the time evolution of each pixel has been approximated by Equation (1) or a similar function, the original data can be reconstructed as follows [12]:

(2) $$T\left(t\right)=exp(\Sigma_{n=0}^{N}a_{n}{\left[ln\left(t\right)\right]}^{n}).$$

The reconstructed sequence in Equation (2) is differentiable and therefore a low-order or a high-order derivative image can be created. On this basis, it is possible to only save the polynomial coefficients regardless of the length of the image sequence. The reconstructed or the derivative images represent any point in time.

It has been found that a 4th-order or a 5th-order polynomial provides the best fit [12]. In this paper, a 5th-order polynomial fitting was used.

The processed data after the above-mentioned approach have an enhanced temporal/spatial resolution and a reduced high frequency noise. Based on these advantages, it has been used as a signal smoothing pre-processing approach, e.g., in [14]. However, in addition to the above features, it can also generate the time-derivative image sequence that can reconstruct the temperature evolution curve. It has been verified to be an effective approach to increase the probability of detection and it can locate a deeper depth [15]. Therefore, in this work, a low-order derivative (1st or 2nd derivative) was used as a pre-processing approach.

It has been known that the impact of fixed artefacts prior to heating plays an important role. The artefacts involve both the surrounding and the camera reflections. Cold image subtraction (CIS) has been verified to be an effective approach to reduce the artefacts by subtracting a cold image or a few averaged images prior to heating [16]. Therefore, refined median filter (RMF) and CIS were also used prior to the 1st or 2nd derivative processing in PT as a pre-processing approach.

### 3.3. Post-Processing Techniques

Principal component thermography (PCT) [17] has been used as an infrared image processing technique to extract the image features and reduce undesirable signals. Different from classical principal component analysis, PCT relies on singular value decomposition (SVD) that extracts spatial and temporal data from a matrix in a compact manner by projecting original data onto empirical orthogonal functions (EOF). Original data can often be adequately represented with only a few EOFs. Usually, an infrared sequence of 1000 images can be replaced by 10 or less EOF [18]. Another advantage of PCT is its suitability to be combined with other image processing techniques, e.g., in [19,20,21].

**Figure 4:**
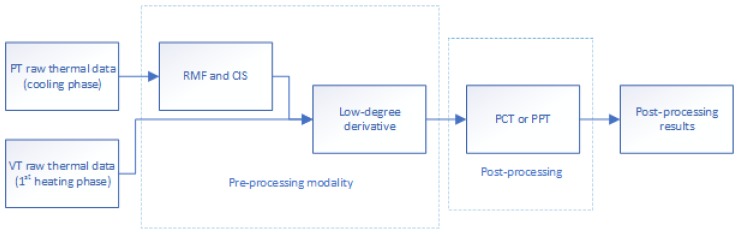
Flow chart of infrared image processing modality.

Pulsed phase thermography (PPT) [22] can be used to extract amplitude and phase information from the raw thermal data. It provides the possibility to obtain quantitative results in a straightforward manner. Usually, phase is more useful than amplitude because it can retrieve the deeper information. In addition, phase is less affected than raw and amplitude data by environmental reflections, emissivity variations, non-uniform heating, surface geometry and orientation [10]. Therefore, PPT was used as a post-processing technique in this work. Figure 4 shows the flow chart of the entire infrared image processing modality.

## 4. Results Analysis

**Figure 5:**
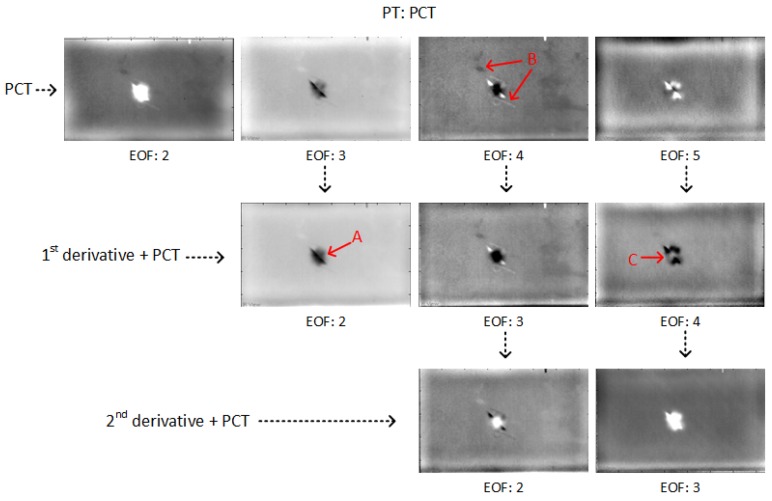
PCT results in PT for CFRP 01.

Figure 5 shows the PCT results in PT for CFRP 01. In the results without pre-processing, more EOFs are required to indicate the defects/features. After pre-processing, less useful EOFs are needed from PCT because the pre-processing reduces the surface and surrounding noise, e.g., surface feature B (marked in red). Therefore, the pre-processing reduces the complexity of the result analysis for PCT. Moreover, the deeper impact damage can be detected more clearly such as A marked in red, and more impact damage (also deeper) can be seen such as C marked in red. Specifically, the 2nd-derivative can reduce the surface/surrounding noise more than the 1st-derivative and less useful EOFs are needed in the results after the 2nd-derivative pre-processing than after the 1st-derivative pre-processing. However, as a pre-processing modality, the 2nd-derivative can also eliminate some useful impact damage information such as C marked in red.

**Figure 6:**
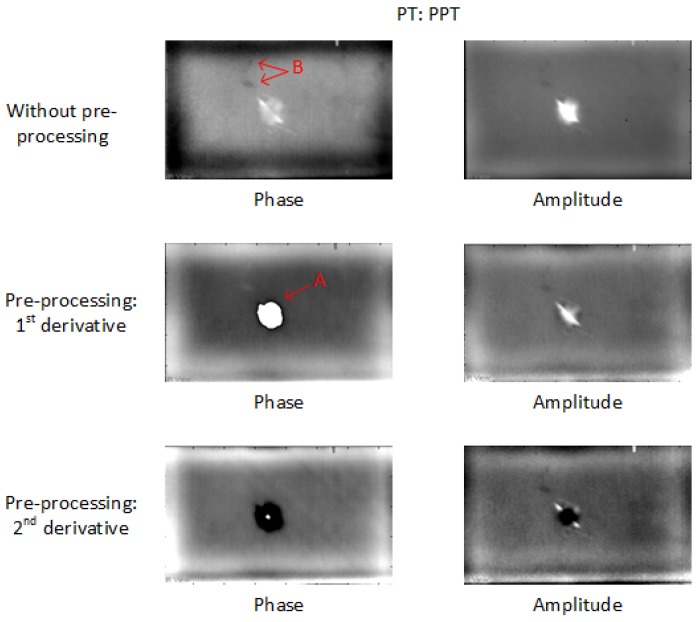
PPT results in PT for CFRP 01.

Compared with PCT, PPT can show a more obvious impact damage area as shown in Figure 6. After the pre-processing, the impact damage (marked A in red) is easier to identify and the surface noise (marked B in red) can be reduced more than in PCT. The phase images show more information than the amplitude images, but they can also lose some useful impact damage information such as A marked in red, which is similar to the results in PCT after the 2nd-derivative pre-processing.

**Figure 7:**
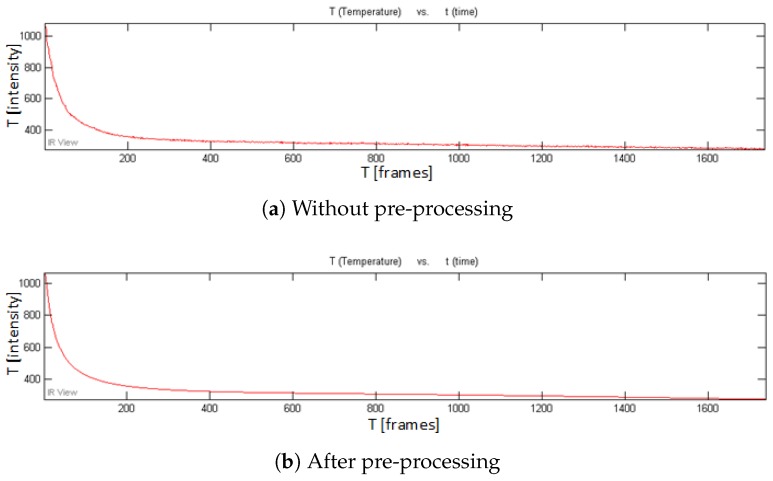
Temperature profile curves (cooling phase) in PT for CFRP 01.

**Figure 8:**
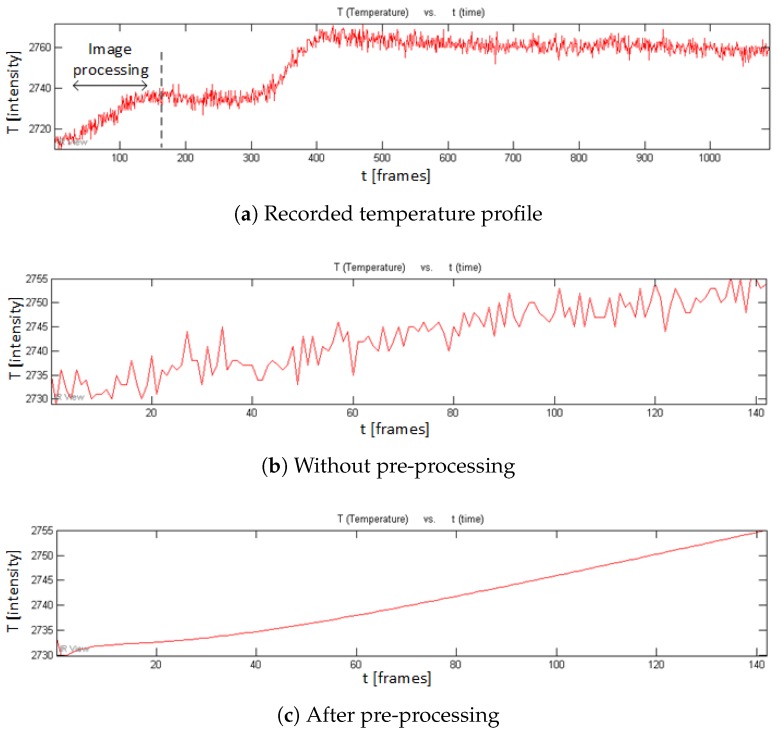
Temperature profile curves in VT for CFRP 01.

**Figure 9:**
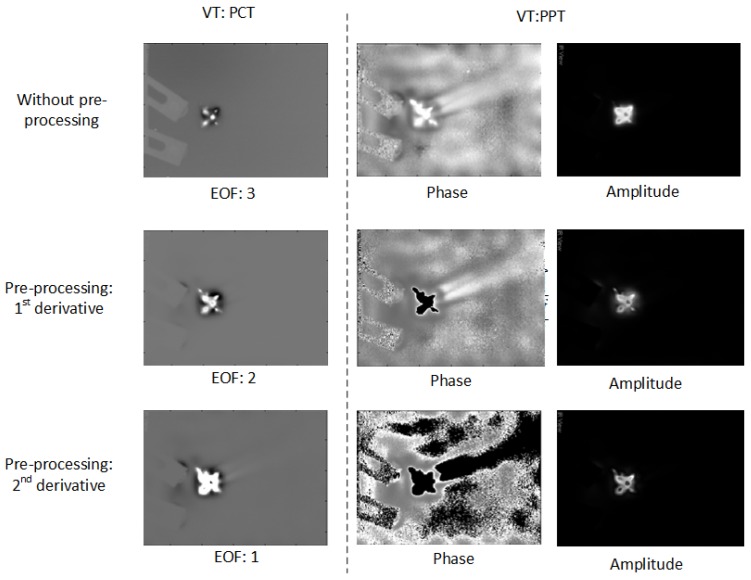
VT results for CFRP 01.

Figure 7 shows the recorded temperature profile curves for a given pixel in a non-defective area in the cooling phase in PT for CFRP 01. It can be seen that the temperature curve is effectively smoothed after the pre-processing in PT, although the effect is not as strong as in the VT curves as shown in Figure 8b,c. Only the 1st heating phase is used for pre-processing (see Figure 8a). A study has shown that more useful defect information can be obtained in the heating phase than in the cooling phase in VT [11]. Because the pre-processing smooths the VT heating phase signals more intensively, the VT results after the pre-processing should show an obvious increased performance in comparison to the results without the pre-processing. This prediction can be verified in the VT results as shown in Figure 9.

Figure 9 shows the VT results for CFRP 01. In the PCT results, a larger impact damage area can been seen after the pre-processing along with the increase of the derivative order. In PPT, the pre-processing can reduce the surrounding noise, but it can also eliminate some useful information similar as in PT.

**Figure 10:**
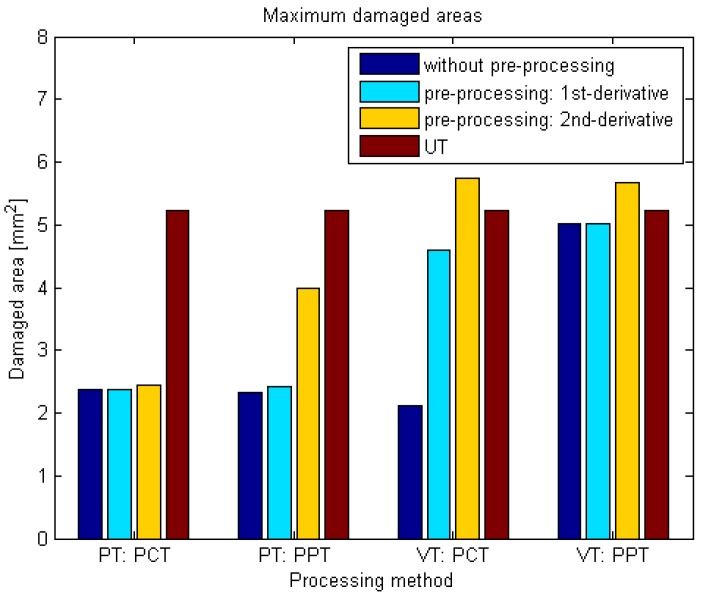
Maximum damaged areas measured in CFRP 01.

Figure 10 shows the maximum damaged areas measured in CFRP 01. The pre-processing modality can detect a larger area than the independent PCT and PPT methods.

**Figure 11:**
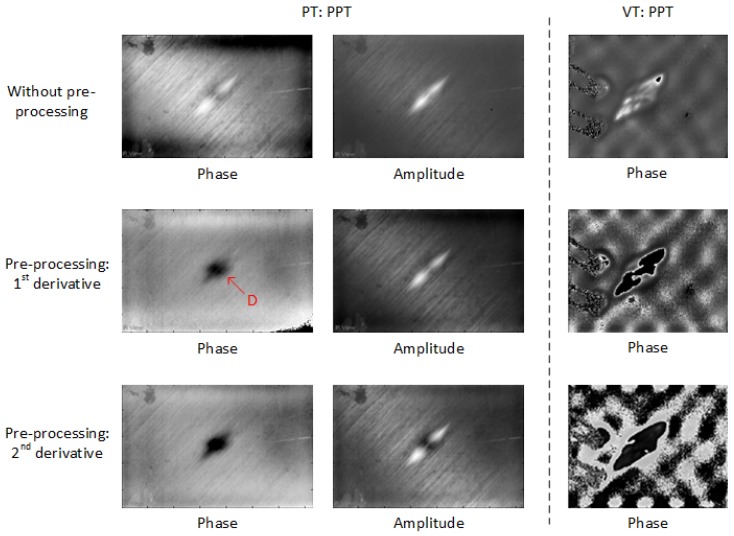
Thermographic results for CFRP 02.

**Figure 12:**
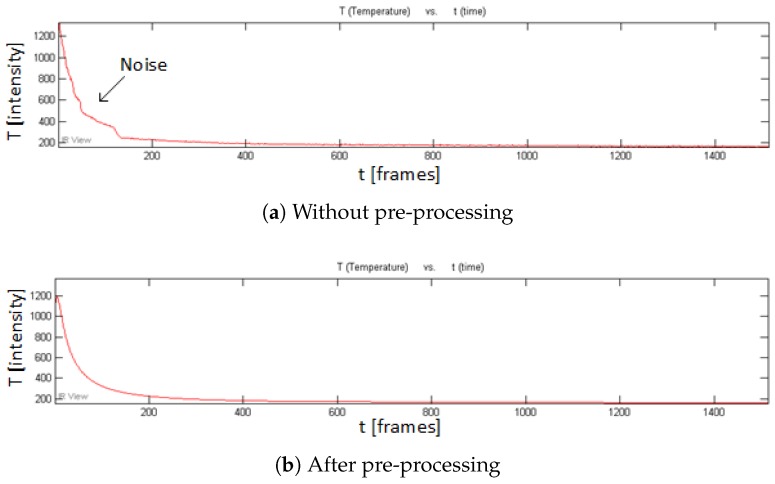
Temperature profile curves (cooling phase) in PT for CFRP 02.

The thermographic results for CFRP 02 show a similar feature as for CFRP 01. In Figure 11, a larger impact area (marked D in red) can be seen in PPT after the pre-processing. The processing effect in CFRP 02 is more obvious than in CFRP 01 because more noise is reduced by the pre-processing as shown in Figure 12.

**Figure 13:**
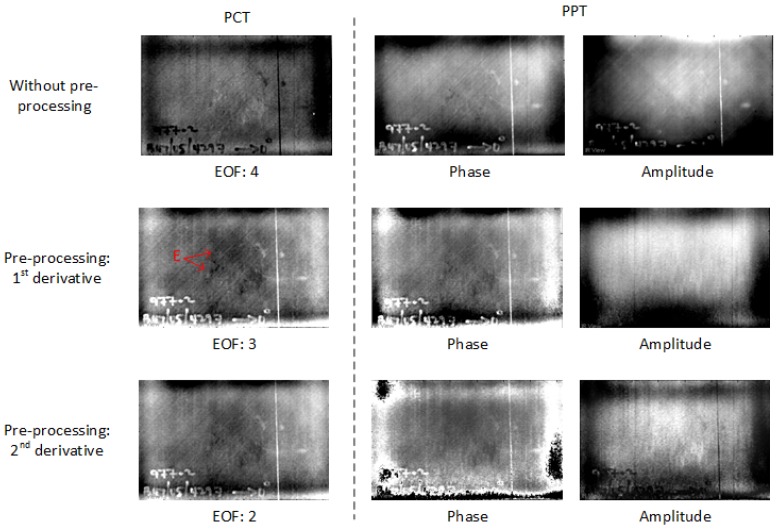
Thermographic results in PT for CFRP 03.

In the thermographic results for CFRP 03 (Figure 13), the resin/preform abnormalities (marked E in red) are shown more clearly in the results after the pre-processing. These features can be verified by the UT results in Figure 2. Indeed, the pre-processing enhances the performance of these inspected features significantly for the case of CFRP 03. In fact, these features are almost invisible in the results without the pre-processing as shown in Figure 13. In addition, VT cannot provide useful information for this type of defect or feature.

**Figure 14:**
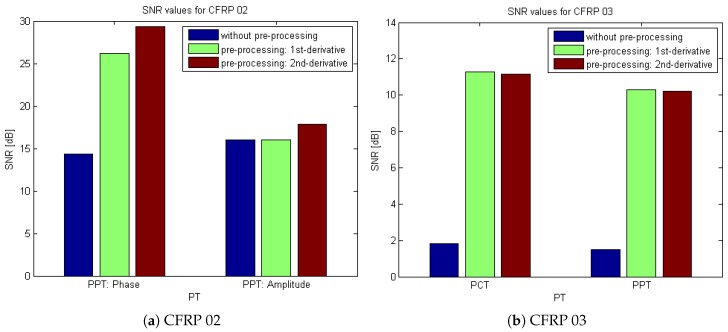
SNR values for CFRP 02 (impacted region) and CFRP 03 (region E) in PT.

Figure 14 shows the signal-to-noise ratio (SNR) values for CFRP 02 (impacted region) and CFRP 03 (region E) in PT. The SNR is expressed in decibels (dB) following the 20 log rule often used in imaging applications, as follows:

(3) $$\mathrm{SNR}=20\cdot \log_{10}|\frac{S_{d}-S_{a}}{\sigma_{Sa}}|$$

where $S_{d}$ represents the intensity of signals, $S_{a}$ represents the intensity of sound areas, and $\sigma_{Sa}$ is the standard deviation representing noise variability [6].

SNR values provide important information about the sensitivity of each technique when comparing the detectability of similar features or defects. Therefore, SNR analysis is used herein as a means to evaluate and compare the sensitivity of the applied processing techniques [6]. Obviously, the SNR values in Figure 14 validate the effect of the pre-processing modality.

**Figure 15:**
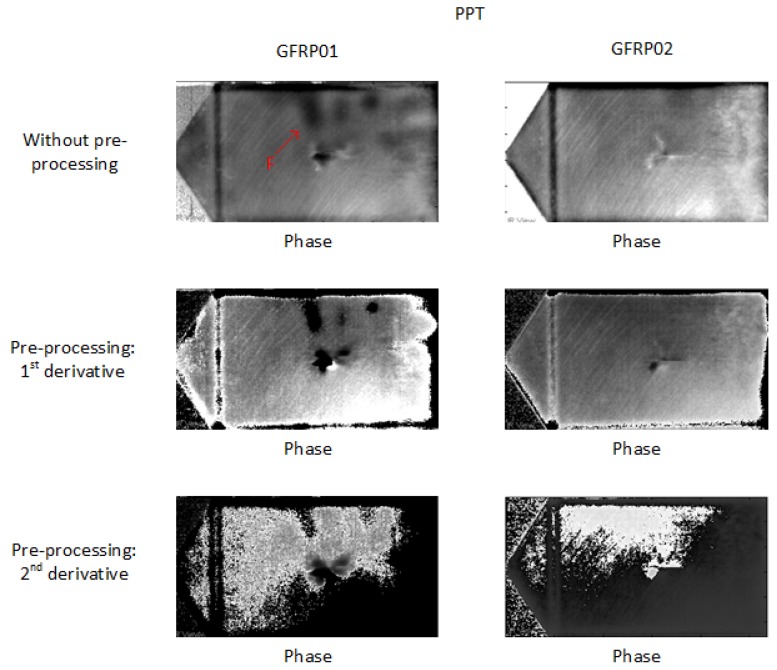
Thermographic results in PT for GFRPs.

The pre-processing does not provide more defect or feature information in PT for GFRPs as shown in Figure 15. The deeper impact damage cannot be seen in the thermographic results and the results after 2nd-derivative pre-processing are foggy. This is probably due to the fact that the GFRP samples are semi-transparent; hence, some infrared radiations pass through the specimen and another part actually heats the surface. Further tests should require the sample to be painted. In addition, GFRP is a bad conductor of heat compared to CFRP, so heat propagates more difficulty [23]. Still, a defect/feature F (marked in red) can be seen, which is not detected by UT (see Figure 2). It might be caused by the shallow-depth resin abnormality, which is not flat so that it does not reflect the ultrasonic signals. However, it cannot be at a deeper depth as explained above. An alternative NDT method such as Terahertz imaging should be more suitable for this type of material [24,25,26,27].

## 5. Conclusions

In this paper, a pre-processing modality was presented for IRT and a few cases were studied involving different defects in CFRP and GFRP specimens. Different from a signal smoothing modality which only uses a polynomial fitting as the pre-processing method, the presented modality instead uses the low-order derivatives to pre-process the raw thermal data. Advanced post-processing techniques such as PCT and PPT were used after the pre-processing procedure. This modality enhances the image performance more than simply using a polynomial fitting as the pre-processing modality or even only using the independent derivative processing as the post-processing technique. Then, UT and SNR analysis were performed for validation. Finally, the effectiveness of this modality was verified for both PT and VT. In addition, it also provides a better performance than lock-in heating in PT in which the presented pre-processing modality cannot be used because the thermal curves for lock-in heating are not linear.

## Figures and Tables

**Table 1:** Damaged areas measured in UT.

Specimen	CFRP 01	CFRP 02	GFRP 01	GFRP 02
Damaged area (cm${}^{2}$)	5.224	7.850	9.112	8.323

## Abbreviations

The following abbreviations are used in this manuscript:

CFRP	Carbon fiber-reinforced polymer
GFRP	Glass fiber-reinforced polymer
NDT	Non-destructive testing
IRT	Infrared thermography
UT	Ultrasonic testing
PT	Pulsed thermography
VT	Vibrothermography
PCT	Principal component thermography
PPT	Pulsed phase thermography
CIS	Cold image subtraction
EOF	Empirical orthogonal function
SNR	Signal-to-noise ratio
